# Osteoclast Fusion: Time‐Lapse Reveals Involvement of CD47 and Syncytin‐1 at Different Stages of Nuclearity

**DOI:** 10.1002/jcp.25633

**Published:** 2016-10-19

**Authors:** Anaïs Marie Julie Møller, Jean‐Marie Delaissé, Kent Søe

**Affiliations:** ^1^ Department of Clinical Cell Biology Vejle Hospital/Lillebaelt Hospital Institute of Regional Health Research University of Southern Denmark Vejle Denmark

## Abstract

Investigations addressing the molecular keys of osteoclast fusion are primarily based on end‐point analyses. No matter if investigations are performed in vivo or in vitro the impact of a given factor is predominantly analyzed by counting the number of multi‐nucleated cells, the number of nuclei per multinucleated cell or TRAcP activity. But end‐point analyses do not show how the fusion came about. This would not be a problem if fusion of osteoclasts was a random process and occurred by the same molecular mechanism from beginning to end. However, we and others have in the recent period published data suggesting that fusion partners may specifically select each other and that heterogeneity between the partners seems to play a role. Therefore, we set out to directly test the hypothesis that fusion factors have a heterogenic involvement at different stages of nuclearity. Therefore, we have analyzed individual fusion events using time‐lapse and antagonists of CD47 and syncytin‐1. All time‐lapse recordings have been studied by two independent observers. A total of 1808 fusion events were analyzed. The present study shows that CD47 and syncytin‐1 have different roles in osteoclast fusion depending on the nuclearity of fusion partners. While CD47 promotes cell fusions involving mono‐nucleated pre‐osteoclasts, syncytin‐1 promotes fusion of two multi‐nucleated osteoclasts, but also reduces the number of fusions between mono‐nucleated pre‐osteoclasts. Furthermore, CD47 seems to mediate fusion mostly through broad contact surfaces between the partners’ cell membrane while syncytin‐1 mediate fusion through phagocytic‐cup like structure. J. Cell. Physiol. 232: 1396–1403, 2017. © 2016 Wiley Periodicals, Inc.

Fusion of osteoclasts (OCs) is an essential step during their differentiation to ensure effective bone resorptive activity. Numerous molecular factors have been identified over the years, which primarily play a major role in the preparative steps required for fusion. The most prominent of these is DC‐STAMP (dendritic cell‐specific transmembrane protein) (Kukita et al., 2004; Yagi et al., 2005; Iwasaki et al., 2008; Mensah et al., 2010; Chiu et al., 2012; Chiu and Ritchlin, 2016), which has been identified as one of the most essential single factors supporting both differentiation and fusion. However, also other factors such as CD47 (Han et al., 2000; Lundberg et al., 2007; Maile et al., 2011; Koskinen et al., 2013; Hobolt‐Pedersen et al., 2014), syncytin‐1 (Soe et al., 2011), OC‐STAMP (osteoclast stimulatory transmembrane protein) (Miyamoto et al., 2012; Witwicka et al., 2015), dynamin (Shin et al., 2014; Verma et al., 2014), Pin1 (peptidyl‐prolyl cis‐trans isomerase NIMA‐interacting 1) (Islam et al., 2014; Cho et al., 2015), and e‐cadherin (Mbalaviele et al., 1995; Fiorino and Harrison, 2016) are involved in OC fusion, but it is important to stress that this list is not exhaustive. In order to identify the role of these factors**,** a series of molecular techniques, and cellular model systems have been employed, which in general are evaluated through end‐point measurements by counting the number of multi‐nucleated OCs, number of nuclei per OC, resorptive activity, and so forth at the end of the incubation period. These analyses are powerful, but are not able to elucidate on the details of individual fusion events leading to this outcome. However, recently analyses of OC fusion in real‐time have been used to identify novel details with regard to the individual fusion partners, their molecular‐ and fusion‐characteristics (Takito and Nakamura, 2012; Takito et al., 2012; Levaot et al., 2015; Soe et al., 2015; Wang et al., 2015; Fiorino and Harrison, 2016). We have recently published a study (Soe et al., 2015), in which we analyzed a large quantity of time‐lapse recordings made over a period of 4 days. Using this technique we showed that multi‐nucleated OCs preferred to fuse with mono‐nucleated cells, that fusion occurred primarily through a broad contact surface or a phagocytic‐cup like structure and that fusion mostly occurred between fusion pairs with divergent motility (Soe et al., 2015).

Previously, we reported that CD47 was primarily expressed in small preOCs/OCs containing few nuclei and that inhibiting CD47 through a blocking antibody primarily reduced the relative presence of OCs with 2–5 nuclei while those with six nuclei or more increased relative to the total number of OCs formed in the presence of CD47 blocking antibody (Hobolt‐Pedersen et al., 2014). In contrast, inhibition of syncytin‐1 mediated cell fusion resulted in a relative increase of preOCs with two nuclei while the proportion of OCs in the culture with six nuclei or more was markedly reduced (Soe et al., 2011). This suggested that CD47 may be involved in early fusion events and syncytin‐1 rather in later events. However, since analyses were done at the end of the culture, this remained a speculation. In order to test the hypothesis that CD47 and syncytin‐1 are truly involved in different stages during OC maturation, we have in the present study analyzed the effect of blocking CD47 and syncytin‐1 function through the analyses of time‐lapse recordings. This allowed us to investigate each single fusion event and the characteristics of the fusion pairs. Our data clearly demonstrate that CD47 promotes cell fusions involving mono‐nucleated preOCs, while syncytin‐1 promotes fusion of two multi‐nucleated OCs, but also reduces the number of fusions between mono‐nucleated preOCs. Furthermore, CD47 seems to mediate fusion mostly through broad contact surfaces between the partners’ cell membrane while syncytin‐1 mediate fusion through phagocytic‐cup like structure.

## Materials and Methods

### Cell culture

CD14^+^ monocytes were isolated from blood of human donors (approved by the local ethical committee, 2007–2019, and written consent was obtained from each donor) by centrifugation through Ficoll–Paque (Amersham, GE Healthcare, Little Chalfont, UK), subsequently suspended in 0.5% BSA and 2 mM EDTA in PBS and were purified using BD IMag™ Anti‐Human CD14 Magnetic Particles −DM (BD Biosciences, San Jose, CA) according to the instructions by the supplier.

CD14^+^ cells were seeded in culture flasks (Greiner, Frickenhauser, Germany) supplied with αMEM (Invitrogen, Taastrup, Denmark) containing 10% FCS (Biological Industries, Kibbutz Beit–Heamek, Israel) and 25 ng/ml human macrophage colony‐stimulating factor (M‐CSF) (R&D Systems, Abingdon, UK) and cultured at 37°C in 5% CO_2_ in a humidified atmosphere (Soe and Delaisse, 2010). After 2 days culture period, the cells were reseeded into eight‐wells of a Nunc Lab‐Tek II chambered cover‐glass (Nunc−Thermo Fisher Scientific, Roskilde, Denmark) at a density of 1.0 × 10^5^ cells/well in αMEM, 10% FCS, 25 ng/ml M‐CSF and 25 ng/ml human receptor activator of nuclear factor kappa‐B ligand (RANKL) (R&D Systems, Minneapolis, MN) (Soe et al., 2015).

After 3 days with M‐CSF and RANKL, the OCs reached an early fusion stage (identified by light‐microscopy) and time‐lapse recordings were initiated. Cells were at this stage supplemented with fresh medium, M‐CSF, RANKL, and supplied with either one of the inhibitors or a corresponding control supplement as described below.

### Time‐lapse recordings and analyses

In the CD47 blocking experiments, cells were cultured with either 1 mg/ml CD47 antibody (clone B6H12; BD, Franklin Lakes, NJ) or with 1 mg/ml mouse IgG1 isotype control antibody (BD) as previously described (Hobolt‐Pedersen et al., 2014). In the syncytin‐1 blocking experiments, cells were cultured with 5 μg/ml syncytin‐1 blocking peptide or with the corresponding concentration of scrambled syncytin‐1‐peptide (KJ Ross‐Petersen, Klampenborg, Denmark) with daily medium change as described in (Chang et al., 2004; Soe et al., 2011).

The chambered cover‐glass was subsequently placed in the incubation chamber of a confocal Olympus Fluoview FV10i microscope (Olympus Corporation, Shinjuku, Tokyo, Japan) with 5% CO_2_ and 37°C for 4 days. Using the software of the microscope, three random sites for each of the eight wells were marked (total of 24 sites), and time‐lapse images were made every 21 min for 23 h using phase contrast. This procedure was repeated for 4 continuous days, and for each new recording three new sites was chosen for each well.

Subsequently, the time‐lapse recordings were analyzed by two observers and the recordings were carefully investigated for fusion events using the FV10‐ASW 4.1/4.2 Viewer software (Olympus). When a fusion event was identified it was characterized by the number of nuclei for each fusion partner and the type of fusion (phagocytic cup, broad contact surface, filopodia/tube or from the top) according to the definitions presented in (Soe et al., 2015). The first observer went through the video material, identified the individual fusion events, marked them on the video, and documented the above‐mentioned details for each fusion event. The second observer verified the marked fusion events, inspected the videos for further events that may have been missed by the first observer and documented the above‐mentioned details separately, without having access to the first observer's documentation. Hereafter, the first observer compared the two observer's data and made the final categorization.

Data was collected from six separate experiments, performed with cells isolated from six different donors. Three experiments were performed for each antagonist. For each data set all four wells per condition for each day were considered as a single data point. Thus, n = 16 for each data set used in the statistical analyses. There were no significant differences in the effects of inhibition between days. For the CD47 blocking experiment, a total of 251 videos reflecting a total of 4,664 h were analyzed; a total of 831 fusion events (control: 483; CD47 antagonist: 348) were observed. For the syncytin‐1 blocking experiment, a total of 235 videos reflecting a total of 5,317 h were analyzed; a total of 987 fusion events (control: 482; syncytin‐1 antagonist: 505) were observed. In order to allow a direct comparison between all experiments, the raw data were converted into the number of fusion events/mm^2^/h and subsequently normalized to the respective control condition.

### Software, data analyses, and statistics

All graphs and statistics were performed using GraphPad Prism software, version 6.07 (GraphPad software, San Diego, CA), and statistical significance was defined as *P *< 0.05. Data sets were tested for normality by using the D'Agostino & Pearson omnibus normality test and this determined whether parametric or non‐parametric tests were used. For the data sets shown in Figure 4 they were interpreted as being normally distributed since medians and means were identical. Outlier analyses were performed using the ROUT method (Q = 0.1%) removing up to a maximum of three data points. Please refer to figure legends for details on the statistical tests used. All experiments were performed three times independently form each other and with cells from different blood donors all with the same outcome. All graphs shown represent data from the same representative experiment.

## Results

### Inhibition of CD47 reduces the total number of fusion events, but inhibition of syncytin‐1 does not

**Figure 1:**
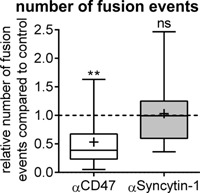
CD47 blockage reduces the total number of fusion events, but blockage of syncytin‐1 does not. Fusions were identified by time‐lapse recording and their number in test conditions are shown normalized relative to that in controls. Analyses were done by combining data from each well (four) and for each day (four) compared to their control. Statistics: Wilcoxon Signed Rank Test testing against a median of 1.0 in the control (indicated with the dashed line). **P < 0.01; ns, not significant. The box‐plot shows the 25–75% quartiles and whiskers indicate the minimum and maximum of the data set. The horizontal line within the box indicates the median and “ + ” indicates the mean, α = antagonist. The graph shown is representative of three experiments for each inhibitor.

When analyzing the total number of fusion events, we found that inhibition of CD47 by a mono‐clonal antibody reduced the total number of fusion events by approximately 50% compared to control condition (Fig. 1) in the presence of an isotype control antibody. In contrast, we observed that inhibition of syncytin‐1 by a specific peptide did not inhibit the total number of fusion events (Fig. 1) compared to a peptide with the same composition of amino acids, but in a scrambled sequence. These very different effects therefore suggest that the modes of action of these two antagonists are different.

### Inhibition of CD47 primarily affects fusion events involving mononucleated preOCs while inhibition of syncytin‐1 selectively inhibits fusion of multinucleated fusion partners

**Figure 2:**
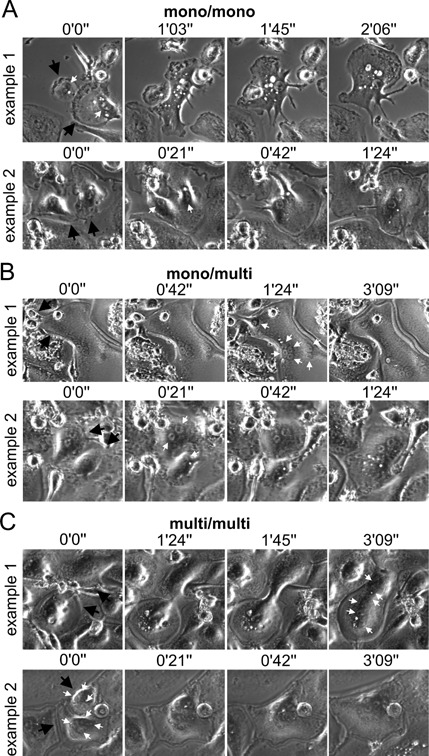
Examples of the different types of fusion pairs used in our analyses. **A**: Two examples of fusion between two mononucleated preOC (mono/mono). **B**: Two examples of fusion between a mononucleated preOC and a multinucleated OC with more than six nuclei (example 1) and two nuclei (example 2) (mono/multi). **C**: Two examples of fusion between two multinucleated OCs (multi/multi). Example 1, fusion between two OCs with three nuclei each; example 2, fusion between an OC with four and an OC with three nuclei. The hours (‘) and minutes (”) between the images in the time‐lapse series are noted above each image. Black arrows point to the fusion pairs in each image series and white arrows point to the nuclei in the fusing cells.

**Figure 3:**
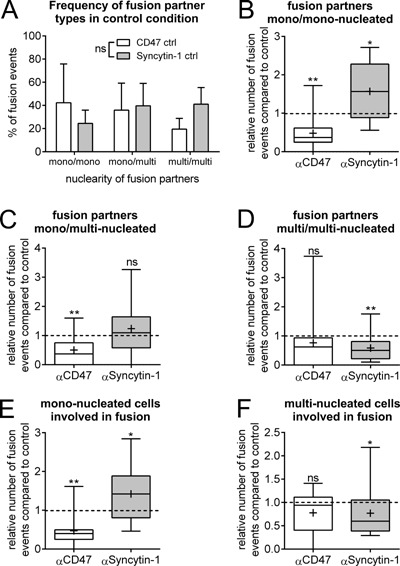
Inhibition of CD47 only affects fusions involving mononucleated preOCs while syncytin‐1 only affects fusion events between multinucleated OCs. Fusion partners identified by time‐lapse recordings were sub‐divided into mono‐ or multinuclear (≥2 nuclei) OCs. **A**: Analysis of the frequency of fusion partners classified according to their nuclearity in two control experiments. The percentage reflects the number of events in a particular category compared to the total number of fusion events for each experiment separately. The graph shows the mean and standard deviation of the results for each well and for each of the days. In **B–F** the fusion frequency involving the different fusion pairs in the presence of inhibitors are shown relative to their respective control. Data are presented and normalized in the same way as described in Figure 1. **B**: Analysis of strictly mononucleated fusion partners. **C**: Analysis of fusion pairs where one was mononucleated while the other was multinucleated. **D**: Analysis of strictly multinucleated fusion partners. **E**: A combined analysis of all fusion events involving mononucleated preOCs. **F**: A combined analysis of all fusion events involving multinucleated OCs. Statistics: In **A**: 2way ANOVA; **B**–**F**: Wilcoxon Signed Rank Test testing against a median of 1.0 in the control (indicated with the dashed line). *, *P *< 0.05; **P < 0.01; ns, not significant. **B**–**F**. The box‐plot shows the 25–75% quartiles and whiskers indicate the minimum and maximum of the data set. Horizontal line within the box indicates the median and “+” indicates the mean, α = antagonist. All graphs shown are representative of three experiments for each inhibitor.

Based on the data shown in Figure 1 it could appear that inhibition of syncytin‐1 does not affect fusion of OCs at all. However, our previous study on OC fusion, also based on time‐lapse recordings (Soe et al., 2015), taught us that it is important to look at the details in order to get a full overview. We therefore analyzed each fusion pair with regard to their nuclearity and categorized them as fusions occurring between two mono‐nucleated OCs (mono<>mono) (Fig. 2A), a mono‐nucleated and multi‐nucleated (2 nuclei or more) OCs (mono<>multi) (Fig. 2B) and two multi‐nucleated OCs (multi < >multi) (Fig. 2C). Figure 3A shows how the distribution of fusion pairs was in control experiments, which matches well data from our previous study (Soe et al., 2015). For the fusion between two mono‐nucleated fusion partners, we found that inhibition of CD47 resulted in a 65% reduction in these fusion events while, surprisingly, inhibition of syncytin‐1 action caused an upregulation by approximately 50% (Fig. 3B). Also fusion between mono‐ and multi‐nucleated partners were suppressed by CD47 antibody by about 65% of the control level while inhibition of syncytin‐1 showed no effect (Fig. 3C). For two multi‐nucleated fusion partners inhibition of CD47 showed no significant effect while syncytin‐1 clearly reduced the number of fusions by 50% (Fig. 3D). Thus, the inhibition of CD47 only affects fusions involving mono‐nucleated preOCs (Fig. 3E) while(whereas) it does not affect multi‐nucleated OCs that fuse (Fig. 3F). On the other hand blocking syncytin‐1 selectively inhibits multi‐nucleated OCs involved in fusion (Fig. 3F) and interestingly it seems to facilitate more fusions of mono‐nucleated preOCs (Fig. 3E). This raises the possibility that syncytin‐1 may facilitate one type of fusion but antagonize another.

**Figure 4:**
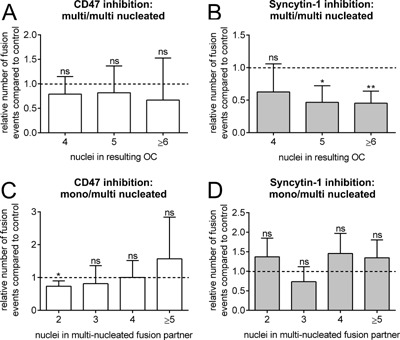
Inhibition of CD47 only affects fusion of mononucleated preOCs with small multinucleated OCs while inhibition of syncytin‐1 primarily affects fusion between OCs with many nuclei. **A** and **B**: The nuclearity of the OCs resulting from fusions between two multinucleated OCs were sub‐divided into those that had 4–6 or more nuclei. The number of fusion events in the presence of inhibitor was normalized to the control condition. **A**: Effect of CD47 inhibition; **B**: Effect of syncytin‐1 inhibition. **C** and **D**: The nuclearity of the multinucleated OCs that fused with a mononucleated preOC was sub‐divided into those where the multinucleated fusion partner had 2–5 or more nuclei. The number of fusion events in the presence of inhibitor was normalized to the control condition and the averages from four wells were used. **C**: Effect of CD47 inhibition; **D**: Effect of syncytin‐1 inhibition. Statistics: One sample t test testing if column means are significantly different from 1.0. *P < 0.05; **P < 0.01; ns, not significant. The graphs shown are representative of three experiments each.

The smallest possible fusion pair, where both partners are multi‐nucleated, is when both have two nuclei resulting in an OC with four nuclei. Thereafter, follows fusion pairs with two and three nuclei to result in an OC with five nuclei, three and three or four and two nuclei both result in an OC with six nuclei and so forth. In Figure 4A and B the multi‐multinucleated fusion pairs were sub‐split according to the nucleation of the fusion product. In Figure 4A it is seen that inhibition of CD47 did not significantly block fusion of any multi‐nucleated fusion pairs whereas inhibition of syncytin‐1 caused significant reductions in those fusion events resulting in OCs with five and six or more nuclei (reduction by 55% in both cases) (Fig. 4B). In the case of fusion between mono‐ and multi‐nucleated partners, CD47 inhibition only resulted in a small reduction (by 27%) of fusions of OCs with two nuclei with mono‐nucleated preOCs (Fig. 4C) while inhibition of syncytin‐1 showed no effect whatever the nuclearity (Fig. 4D).

With regard to the nuclearity of the fusion partner it can be concluded that CD47 is only required when a mono‐nucleated preOCs is involved, while syncytin‐1 specifically supports fusion of multi‐nucleated cells. It is also of interest that syncytin‐1 appears to antagonize fusion between two mono‐nucleated preOCs.

### Inhibition of CD47 and syncytin‐1 differently affects the morphological appearance of fusion partners

**Figure 5:**
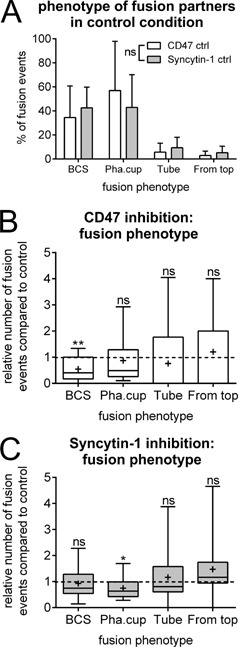
Inhibition of CD47 and syncytin‐1 differently affects the phenotype of fusion partners immediately prior to fusion. The fusion phenotype of fusion partners were sub‐divided into four different phenotypes: broad contact surface (BCS), phagocytic cup (Pha.cup), fusion at the tip of tube‐like structures (Tube) and where one fusion partner sits on top before fusion occurs (From top). **A**: Analysis of the frequency of fusion partners classified according to their fusion phenotype in two control experiments. The percentage reflects the number of events in a particular category compared to the total number of fusion events for each well and each day. The graph shows the mean and SD. **B** and **C**: The percentage reflects the number of events in a particular category compared to the total number of fusion events for each well and each day. The box‐plot shows the 25–75% quartiles and whiskers indicate the minimum and maximum of the data set. Horizontal line within the box indicates the median and “+” indicates the mean. **B**: Effect of CD47 inhibition; **C**: Effect of syncytin‐1 inhibition. Statistics: In **A**: 2way ANOVA; **B** and **C**: Wilcoxon Signed Rank Test testing against a median of 1.0 in the control (indicated with the dashed line). *P < 0.05; **P < 0.01; ns, not significant. All graphs shown are representative of three experiments each.

When analyzing the time‐lapse recordings we have also been able to categorize the morphological appearance of the fusion partners at the site of fusion. As defined in our previous study (Soe et al., 2015), we have categorized fusion phenotypes as: broad contact surface, phagocytic cup, tube or fusion from top (examples of these fusion phenotypes can be viewed in the illustrations and supplementary videos of our prior publication Soe et al. (2015)). Just as we previously reported, broad contact surface, and phagocytic cup are by far the most common fusion phenotypes with 45% and 43% of the fusion events, respectively (Fig. 5A). Fusion through the tip of filopodias/tubes and where one cell “crawls” on top of the fusion partner before fusion occurs (from top) were far less common with only 6.7% and 5.2%, respectively (Fig. 5A). In the presence of CD47 blocking antibody, the frequency of the broad contact surface was specifically reduced by 60% compared to control (set to 1) (Fig. 5B). In contrast, in the presence of syncytin‐1 blocking peptide it was specifically the frequency of fusions occurring through phagocytic cup that were significantly reduced by 37% (Fig. 5C).

## Discussion

In our present study, we have tried to overcome a fundamental problem of research on cell–cell fusion, namely that the vast majority of studies on fusion factors are based on measurements using multi‐nucleated cells as end‐point. Unfortunately, this type of approach only allows for speculation on how this end‐point was reached. But since fusion is a fast process and multinuclearity can be obtained through different intermediate fusion routes, it is very difficult to understand the precise involvement of any relevant fusion‐related factor based on end‐point measurements. Increasing awareness that OC fusion is not a random process and that there is a stringent selection of the fusion partner, apparently based on heterogeneity between partners, makes it necessary to pay special attention to each individual fusion event. We have therefore in our present study tested the hypothesis of heterogenic involvement of different fusion factors at different levels of the OC maturation process. We have chosen to use two antagonists targeting fusions mediated through CD47 and syncytin‐1. These two candidates were chosen because we have previously seen indications that these two factors are active during different stages of OC fusion and maturation (Soe et al., 2011; Hobolt‐Pedersen et al., 2014). Using the time‐lapse approach and these antagonists we now demonstrate that CD47 and syncytin‐1 are indeed involved in different processes during human OC fusion. Blocking of CD47 specifically had an effect on fusion involving mononucleated preOCs in the fusion pair. In contrast, blocking syncytin‐1 mediated fusion only inhibits fusion of two multinucleated OCs resulting in five nuclei or more.

Inhibition or knock‐out of CD47 was previously reported to inhibit fusion of murine OCs (Lundberg et al., 2007; Maile et al., 2011) as well as macrophages (Han et al., 2000) in cell culture. In these studies, the total number of OCs with three nuclei or more was analyzed at the end of the culture, but without reporting details on the actual number of nuclei per cell (Lundberg et al., 2007; Maile et al., 2011; Koskinen et al., 2013). In our recent study (Hobolt‐Pedersen et al., 2014), we used a CD47 blocking‐antibody and evaluated at the end of the culture the number of nuclei in all OCs both in control condition as well as in the presence of the blocking antibody. We found that the CD47 blocking‐antibody reduced the relative frequency of OCs with 2–5 nuclei, but surprisingly we also found a relative increase in the fusion events resulting in six nuclei or more out of the total OCs. However, we also found that preOCs/OCs with few nuclei were strongly positive for CD47 while larger OCs were less so. We therefore proposed that CD47 has a heterogenic expression amongst OCs at various stages of differentiation and that it is primarily involved in early fusion events involving preOCs/OCs with few nuclei (Hobolt‐Pedersen et al., 2014). This is now demonstrated in our present study by additional information that better allows us to interpret the involvement of CD47 in OC fusion. Inhibition of CD47 resulted in: (i) a significant drop in the number of fusion events in real‐time (Fig. 1) in line with prior proposals (Lundberg et al., 2007; Maile et al., 2011); (ii) a strong reduction in fusion events between two mono‐nucleated preOCs; (iii) a specific inhibition of fusion between a preOCs and an OC with two nuclei, but without affecting fusion with OCs containing three nuclei or more; (iv) no effect on fusion between two multi‐nucleated OCs; (v) a specific reduction in fusion events mediated through the broad contact surface phenotype. These data fit the view discussed by Vignery (2005) that CD47 is involved in recognition of “self” and as a possible fusion partner. This issue appears particularly critical for fusions with a mono‐nucleated cell since it is at this level that selection of the correct fusion partner has to be very strict in order to avoid detrimental effects. CD47 may be such a critical hallmark.

In our previous study, an end‐point analysis showed that inhibition of syncytin‐1 surprisingly resulted in a higher relative frequency of OCs with two nuclei while the relative frequency of OCs with six nuclei or more was significantly reduced (Soe et al., 2011). We speculated that the higher number of binucleated OCs resulted from an accumulation of this fusion stage because later fusion steps were blocked. With our present study we are now able to re‐interpret our previous observations. Our new study using time‐lapse recordings clearly shows that inhibition of syncytin‐1 resulted in: (i) a strong increase in the number of fusion events between two mononucleated preOCs; (ii) no effect on fusions between a mononucleated preOC and a multinucleated OC; (iii) a marked inhibition of fusion between two multinucleated OCs in particular when the resulting OC has at least five nuclei; (iv) a specific reduction in fusion events mediated through the phagocytic cup phenotype. There are only very few studies that have addressed the involvement of syncytin‐1 in cell fusion and at the same time stratified for the number of nuclei in the resulting cells. However, Frendo and colleagues (Frendo et al., 2003) did so when investigating the fusion of primary human cytotrophoblasts in the presence or absence of syncytin‐1 anti‐sense RNA. They found, using end‐point analyses, that only the frequency of syncytia with six nuclei or more was significantly reduced, which supports data from our present study. At the same time they also observed that this reduction was compensated by a strong increase in syncytia with 3–5 nuclei. This matches our previous (Soe et al., 2011) as well as our present results. However, the observed increase in the number of fusion events between mononucleated preOCs is surprising. Yet, this finding is not unique to syncytin‐1, but has also been seen in other studies. Shin et al. (2014) reported that a double knock‐out of dynamin one and two inhibited fusion of OCs resulting in cells with seven nuclei or more, but especially the number of OCs with two and three nuclei were actually fourfold more common. Very similar results were reported by Verma and colleagues who also investigated the involvement of dynamins in the fusion of OCs (Verma et al., 2014). Finally, in a study using knock‐outs of OC‐STAMP it was also shown that OC‐STAMP deficiency strongly reduced fusion of OCs resulting in three nuclei or more, but apparently the fusion of mononucleated preOCs still went on since there was a clear accumulation of bi‐nucleated OCs (Witwicka et al., 2015). Although none of these studies used time‐lapse analyses to accurately observe the effect of knock‐outs, anti‐sense RNA or inhibition on individual fusion events, their results are similar to those we obtained in our original study inhibiting syncytin‐1 fusion (Soe et al., 2011). Although others have reported similar results it is uncertain how this unexpected result may be explained and more knowledge is needed based on research tools like time‐lapse recordings. Since only end‐point measurements have been used previously it has been interpreted as an accumulation of small OCs because their fusion was unaffected by treatment or because it caused defects in cytokinesis during the cell cycle (Soe et al., 2011; Shin et al., 2014; Witwicka et al., 2015) and therefore more OCs with few nuclei accumulated. However, only if real‐time analyses are used all possibilities can be clearly distinguished and in our study we can rule out that it was due to a defect in cytokinesis or accumulation of cells since this can clearly be addressed using time‐lapse. Whatever the explanation, it is interesting that observations based on several fusion factors point to a model where some “fusion factors” specifically favor the addition of more nuclei to pre‐existing multinucleated cells and hinder the generation of additional multinucleated cells.

**Figure 6:**
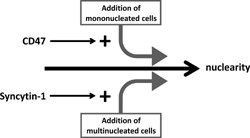
Schematic representation of the overall involvement of CD47 and syncytin‐1 in OC fusion. Our data have prompted us to suggest this model on the specific involvement of CD47 and syncytin‐1 in OC fusion. Please refer to text in the Discussion for details.

In Figure 6 we show a model of how our data may be summarized in the context of facilitating and regulating OC fusion. CD47 primarily contributes to OC fusion by facilitating the formation of numerous but small OCs mostly with two nuclei, but also facilitating a slowly increasing number of nuclei in OCs by adding one nucleus at a time. In contrast, syncytin‐1 contributes to reducing the number of small multinucleated OCs by facilitating their fusion and therefore generating large OCs with many nuclei, but at the expense of less OCs. The latter effect is even more pronounced since we also found that syncytin‐1 seems to antagonize fusion between mononucleated cells in the first place. One may speculate that this antagonistic effect results from a negative effect of syncytin‐1 on OC migration (Mo et al., 2013). Alternatively, it may also involve receptor interference as known from virus preventing a host cell from fusing with other viruses (Kjeldberg et al., 2011). Whatever the explanation, physiologically, it may make sense that multinucleated cells form initially, but that their size and number needs to be controlled. The involvement of factors only at certain steps may present a possibility to control the potentially deleterious fusion of cells. This goes for OCs but certainly also for example the syncytiotrophoblast and muscle.

Finally, in accordance with our previous findings (Soe et al., 2015) the present study shows that the most common fusion morphologies of fusion pairs were what we have called broad contact surface and phagocytic cup (Fig. 5A). We found that there were small but significant effects on the frequency of these fusion morphologies between the two treatment types. In the case of CD47 inhibition we found a significant reduction in the frequency of fusions through broad contact surface (Fig. 5B), while we found a reduction in the frequency of phagocytic cup in the case of syncytin‐1 inhibition (Fig. 5C). Although recently a number of studies have focused on the morphologies of fusion partners immediately prior to fusion (Oikawa et al., 2012; Takito and Nakamura, 2012; Takito et al., 2012; Fiorino and Harrison, 2016), knowledge about the molecular composition at these fusion sites is still very scarce. However, considering that syncytin‐1 originates from a retroviral fusion protein it may make sense that it is this fusion route that is affected. It is known that syncytin‐1 originates from a family of retroviruses, which are suggested to fuse with host cells through for example phagocytosis/endocytosis (Permanyer et al., 2010; Lokossou et al., 2014). With regard to the effect of CD47 inhibition on fusions through the broad contact surface, it is more difficult to interpret because of the very limited knowledge about CD47's role in cell‐cell fusion. However, it is known that CD47 through strictly regulated clusters on the extracellular membrane (Ha et al., 2013), is part of signaling pathways preventing cells from being phagocytized by macrophages.

## Conclusion

In conclusion, the present study demonstrates that the respective roles of CD47 and syncytin‐1 in OC fusion differ depending on the degree of nuclearity of the fusion partners. Overall, it suggests that fusion of OCs is not just the same process from the very first fusion between two mononucleated preOCs to the fusion between large multinucleated OCs. Our observations have lead to a model where the molecular mechanism of cell‐cell fusion is evolving during the successive fusion of cells leading to a given number of nuclei per cell. These findings have important methodological implications, since they highlight that the role of a given fusion factor has to be evaluated specifically for each fusion step leading to the multinucleated cell. It cannot be properly evaluated by analyzing only the resulting multinucleated cell as end‐product—which otherwise is the common approach. We would therefore like to advocate for more studies using approaches such as quantitative analyses of time‐lapse recordings in order to generate the necessary critical mass of studies to unravel how cell‐cell fusion is regulated and controlled.
